# Low breast milk levels of long-chain n-3 fatty acids in allergic women, despite frequent fish intake

**DOI:** 10.1111/j.1365-2222.2010.03678.x

**Published:** 2011-04

**Authors:** S Johansson, A E Wold, A-S Sandberg

**Affiliations:** 1 Food Science, Department of Chemical and Biological Engineering, Chalmers University of TechnologyGöteborg, Sweden; 2 Department of Infectious medicine, Clinical Bacteriology Section, Institute of Biomedicine, University of GothenburgGöteborg, Sweden

**Keywords:** allergy, atopic eczema, atopy, breast milk, DHA, EPA, fatty acids

## Abstract

**Background:**

Long-chain n-3 polyunsaturated fatty acids (PUFAs) have immune regulating and anti-inflammatory effects. However, their role in allergic disease is unclear. Allergic diseases are immunologically heterogeneous, and we hypothesized that n-3 fatty acid composition in serum and breast milk may vary according to clinical manifestations. Further, animal studies have shown reduction of serum-PUFA levels during allergic inflammation.

**Objective:**

To investigate fatty acid composition in breast milk and serum from women with different atopic disease manifestations. Secondly, to determine whether low PUFA levels reflected insufficient intakes.

**Methods:**

Fatty acids were analysed in breast milk and serum of women with *atopic* eczema and respiratory allergy (*n*=16), only respiratory allergy (*n*=7), as well as healthy women (*n*=22). Dietary intake of foods expected to affect long-chain n-3 PUFA levels were estimated by food-frequency questionnaire. The fatty acid pattern was related to diagnostic group and intake of relevant food items using a multivariate pattern recognition method (partial least squares projections to latent structures and discriminant analysis).

**Results:**

Women with a combination of eczema and respiratory allergy had lower breast milk levels of several PUFAs (arachidonic acid, eicosapentaenoic acid, EPA, docosahexaenoic acid, DHA, and docosapentaenoic acid, DPA), and a lower ratio of long-chain n-3 PUFAs/n-6 PUFAs. Their PUFA levels differed not only from that of healthy women, but also from that of women with only respiratory allergy. The latter had a fatty acid pattern similar to that of healthy women. Despite low EPA, DHA and DPA levels women with eczema and respiratory allergy consumed no less fish than did healthy women.

**Conclusion & Clinical Relevance:**

Our data suggest that reduced levels of long-chain n-3 fatty acids in serum and breast milk characterize women with extensive allergic disease including eczema, and are not related to low fish intake. Consumption of PUFAs during the allergic process may explain these findings.

Allergies have increased dramatically in Western societies during the last century [1]. Factors associated with the Western life-style, such as reduced exposure to microbes, have been proposed to explain this epidemic [2]. In addition, both epidemiological and experimental evidence suggest that dietary habits may influence the risk of developing allergy [3]. Dunder et al. [4] proposed in 2001 that increased intake of unsaturated fatty acids could increase the risk of allergy development as the diet of children with atopic dermatitis contained less butter and more margarine and, hence, was characterized by a higher ratio of polyunsaturated/saturated fatty acids than of healthy controls. Similarly, von Mutius et al. [5] reported that consumption of less butter and more margarine could explain the rise in atopy with Westernization. Margarine contains high concentrations of the n-6 polyunsaturated fatty acid (PUFA) linoleic acid (LA) compared with butter, which mainly contains saturated fat [5].

Other types of long-chain PUFAs, mainly of the n-3 series, are found in fish. These include eicosapentaenoic acid (EPA), docosahexaenoic acid (DHA) and docosapentaenoic acid (DPA). Consumption of n-3 PUFAs may modulate various immunologic and inflammatory pathways [6]. Recent publications have reported lower incidence of eczema in children whose diet has included fish [7–9] and whose mothers had high fish intake during pregnancy [10, 11]. Consumption of fish has been associated with reduced allergic symptoms also in adults [12, 13].

It is generally considered that dietary intake of fish n-3 fatty acids is reflected in breast milk and serum. But EPA, DPA and DHA can also be synthesized by humans from α-linolenic acid, a shorter type of n-3 PUFA found in green leafy vegetables and vegetable oils. However, EPA and DPA are synthesized at low efficiency and DHA seems to increase very little with α-linolenic acid supplementation [14]. Intervention with long-chain n-3 PUFAs have yielded conflicting results, some studies reporting reduced allergic symptoms [15, 16], others no effect [17]. Accordingly, high n-3 PUFA content of mother's milk has been associated with lower [18, 19] or higher [20] risk of allergy development in the infant. Still another study shows no relation to allergy but reduction of non-atopic eczema [21]. Long-chain n-3 PUFA content in breast milk from atopic and non-atopic women have also been compared in several studies, with conflicting results. Some showed no fatty acid differences [20, 22, 23], some reported lower n-3 fatty acid levels [24, 25] and one [26] higher DPA levels in milk from atopic compared with healthy mothers. Studies regarding PUFA content in breast milk have usually included women with different atopic manifestations, such as asthma, hayfever and/or eczema. One reason for the divergent results regarding n-3 PUFA pattern in atopy may be that these mediators have different effects on different allergic manifestations, e.g. asthma vs. atopic eczema.

A further complication is that low serum PUFA levels are associated with active allergy, this may either indicate a protective effect of n-3 fatty acids, or enhanced consumption of these fatty acids during inflammation. Evidence supporting an enhanced consumption of long-chain n-3 PUFAs includes Dunder's et al. [4] study in which children with atopic eczema were found to have lower serum levels of EPA and DHA than non-atopic children, despite similar levels of fish consumption. Furthermore, we have recently demonstrated a pronounced consumption of long-chain PUFAs, including EPA and DHA during the challenge phase of an experimental airway allergy model [27].

The aim of this study was to investigate differences in fatty acid composition in breast milk and serum from healthy women and women suffering from atopic eczema and/or respiratory allergy. The second aim was to investigate whether differences in PUFA levels correlated with differences in dietary intake.

## Methods

### Subjects

The study population comprised 45 women who gave birth from September 2005 to September 2007, 18 of whom were from the city of Göteborg in southwest Sweden and 27 living in a rural area around 100 km from Göteborg. The women were recruited at the respective antenatal clinics and filled out a questionnaire probing for doctor's diagnosed allergic disease. Women with a clear history of asthma, allergic rhinitis (AR) and/or atopic eczema were included, as well as a number of healthy women without any allergic symptoms. To confirm the atopic/healthy state, a blood sample was drawn and analysed for total IgE and specific IgE antibodies to a panel of common inhalant allergens, namely: dog, cat, horse, birch, timothy, mugwort, *Dermatophagoides pteronyssinus, D. farinae* and *Cladosporium* (Phadiatop, Pharmacia, Uppsala, Sweden). The analyses were performed at the Allergy Laboratory, Sahlgrenska University Hospital, Göteborg, Sweden.

The groups were of similar age (median age 32, 32 and 30 years for healthy, eczema+respiratory allergy and respiratory allergy only, respectively). Only two women reported smoking (one healthy, one eczema) and two women in the eczema group reported intake of omega-3 supplements (one fish oil and one flaxseed oil). In the eczema group 13 of 16 women took allergy medication (eight antihistamines, five β_2_-agonists, six local cortisone, one sodium cromoglicate and one combined corticosteroids and β_2_-agonists). In the respiratory group, four of seven women took medication (four antihistamines, one β_2_-agonists, one systemic cortisone, one local cortisone and one sodium cromoglicate).

The participating women were asked to complete a food-frequency questionnaire based on the validated Northern Sweden FFQ [28], including selected foods expected to affect long-chain n-3 and n-6 fatty acid levels in breast milk and serum, i.e. marine foods (fat fish, lean fish and shellfish), butter, margarine and vegetable oils. The participants were asked to mark how often they consumed different food products, and the frequencies were then converted to g/wk. Written informed consent was obtained and the study was approved by the regional Ethics Committee, University of Gothenburg, permission number 363-05.

### Collection of breast milk and serum samples

Breast milk samples were collected 1 month post-partum, during the second meal of the day. Five to 10 mL of milk was expressed manually into sterile plastic tubes and frozen immediately at –20 °C at home. Milk samples were later transported to the laboratory for storage at –80 °C until analysed. Venous blood, 5–10 mL, was drawn from the women and after centrifugation, serum was immediately frozen in aliquots and stored at –80 °C until analysed.

### Analysis of fatty acids in breast milk

**Fig. 1:**
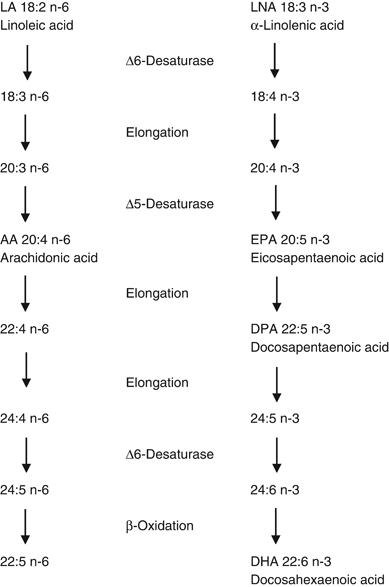
The metabolic pathway for polyunsaturated fatty acids linoleic and α-linolenic acids.

Fatty acids in the total lipid fraction of breast milk samples were analysed by gas chromatography after conversion to methyl esters [29]. Milk samples (100 μL) were thawed slowly in cold water, vortexed and mixed with 2 mL toluene, 2 mL acetyl chloride (10%) dissolved in methanol, and 50 μL internal standard (fatty acid 21:0, 0.5 mg/mL). After incubation in a 70 °C water bath for 2 h, methyl ester were extracted with petroleum ether, and after evaporation, dissolved in isooctane and separated by gas chromatography (Hewlett Packard 5890, Waldbronn, Germany) on a HP Ultra 1 (50 m × 0.32 mm × 0.52 μm DF) column (J&W Scientific, Folsom, CA, USA). Detection was carried out by flame ionization and the Borwin software (JMBS Developments, Le Fontanil, France) was used for evaluation. The samples were analysed regarding the 12 unsaturated fatty acids shown in Fig. 1 with a length of 18–22 carbon atoms, as well as 10 other fatty acids; all shown in Table 1. The n-3 fatty acids EPA (20:5 n-3), DPA (22:5 n-3) and DHA (22:6 n-3) are characteristically present in fish, but can also be formed from α-linolenic acid (LNA, 18:3 n-3), Fig. 1. Long-chain n-6 fatty acids can be formed from dietary LA (18:2 n-6) by elongation (Fig. 1).

**Table 1:** Fatty acid content in the total lipid fraction of breast milk (1 month)

	Fatty acids (% of total fatty acids)			
		
	Healthy (*n*=22)	Eczema+respiratory allergy (*n*=16)	Only respiratory allergy (*n*=7)	*P*-value
18:0	7.3 ± 0.26	7.0 ± 0.55	6.9 ± 0.40	NS
18:1 n-9	37 ± 0.57	34 ± 2.2*	39 ± 0.58*	0.03
18:1 n-7	2.2 ± 0.08	1.9 ± 0.25	2.2 ± 0.08	NS
18:2 n-6 (LA)	10 ± 0.42	9.6 ± 0.87	10 ± 0.55	NS
18:3 n-6	0.15 ± 0.01	0.23 ± 0.07	0.17 ± 0.04	NS
18:3 n-3 (LNA)	1.4 ± 0.07	1.3 ± 0.15	1.3 ± 0.10	NS
18:4 n-3	0.05 ± 0.01	0.11 ± 0.03	0.06 ± 0.02	NS
20:4 n-6 (AA)	0.41 ± 0.02	0.37 ± 0.02*	0.45 ± 0.02*	0.01
20:5 n-3 (EPA)	0.14 ± 0.01*^,^^a^	0.10 ± 0.01*^,^^a^^,^^b^	0.15 ± 0.02*^,^^b^	0.05^a^, 0.02^b^
20:3 n-6	0.40 ± 0.02	0.41 ± 0.02	0.38 ± 0.03	NS
20:4 n-3	0.22 ± 0.01	0.20 ± 0.01	0.18 ± 0.02	NS
20:2 n-6	0.26 ± 0.01	0.29 ± 0.02	0.26 ± 0.01	NS
20:1 n-7	0.055 ± 0.003	0.062 ± 0.004	0.051 ± 0.001	NS
20:0	0.20 ± 0.01	0.22 ± 0.01	0.20 ± 0.01	NS
22:5 n-6	0.030 ± 0.002	0.025 ± 0.002	0.029 ± 0.003	NS
22:6 n-3 (DHA)	0.33 ± 0.04	0.24 ± 0.03*	0.34 ± 0.04*	0.04
22:4 n-6	0.072 ± 0.004	0.071 ± 0.004	0.069 ± 0.003	NS
22:5 n-3 (DPA)	0.19 ± 0.01^a^	0.16 ± 0.01^a^^,^^b^	0.19 ± 0.01^b^	0.05^a^ (*P*=0.06^b^)
22:2 n-6	0.07 ± 0.03	0.04 ± 0.003	0.04 ± 0.01	NS
22:1 n-9	0.11 ± 0.01	0.098 ± 0.004	0.091 ± 0.01	NS
22:3 n-3	0.007 ± 0.001	0.006 ± 0.001	0.003 ± 0.002	NS
22:0	0.09 ± 0.004	0.14 ± 0.04	0.081 ± 0.01	NS
LA/LNA	7.6 ± 0.39	7.7 ± 0.52	8.4 ± 0.59	NS
AA/EPA	3.3 ± 0.21^a^	3.8 ± 0.20^a^^,^^b^	3.2 ± 0.34^b^	NS (*P*=0.06^a^, *P*=0.07^b^)
AA/DHA	1.6 ± 0.15	1.7 ± 0.13	1.4 ± 1.2	NS
AA/DPA	2.3 ± 0.11	2.5 ± 0.12	2.4 ± 0.18	NS
n-6 LCP/n-3 LCP	1.5 ± 0.07*^,^^a^	1.8 ± 0.09*^,^^a^^,^^b^	1.4 ± 0.10*^,^^b^	0.03^a^, 0.02^b^
tot n-6/tot n-3	4.9 ± 0.49^a^^,^^b^	6.1 ± 0.44^a^	6.5 ± 0.36^b^	NS (*P*=0.09^a^, *P*=0.07^b^)

* These groups are significantly different, *P*≤0.05.

a,b Marking of group-differences with *P*-value as indicated.

ns, no significant group differences; LCP, long chain polyunsaturated fatty acids, the sum of all fatty acids analysed, except the 18 carbon long fatty acids; tot, total fatty acids, the sum of 14–22 carbon atoms long fatty acids.

### Analysis of serum fatty acids

Serum phospholipid composition was determined by extracting fat from serum with chloroform and methanol [30]. Serum (500 μL) was mixed with 4 mL of chloroform: methanol (1:2), 2 mL 0.5% NaCl-solution and 50 μL of the internal standard, fatty acid 17:0. The chloroform phase was collected, and after evaporation, the fat was dissolved in 200 μL chloroform. Phospholipids were obtained by separation on aminopropyl solid phase extraction columns (Isolute NH_2_, 6 mL, 500 mg, IST, Mid Glamorgon, UK) [31]. After evaporation, phospholipids were redissolved in 1 mL toluene, converted to methyl esters and analysed as described above.

### Total content of protein and lactose in breast milk

Total protein concentrations in milk samples were determined spectrophotometrically according to Lowry [32] as modified by Markwell [33]. The lactose content was determined by high-performance liquid chromatography [34].

### Statistical analysis

Principal component analysis (PCA) and projections to latent structures by means of partial least square (PLS) are two multivariate data analysis techniques particularly suitable when having large data sets consisting of numerous variables. In PCA, the original set of correlated variables are combined into a small number of new variables, termed ‘latent variables’ or ‘principal components’, each of which accounts for as much of the variance of the original data as possible and which are also uncorrelated to one another [35]. In PLS, ‘latent variables’ or ‘score vectors’ that covaries maximally with a selected Y variable, for example a certain clinical condition, is searched, which makes PLS a regression extension of PCA [36]. PLS with discriminant analysis (PLS-DA) separates observations into predetermined classes of observations (e.g. different clinical conditions) on the basis of all variables [35]. The variables that contribute to the separation of the classes can then be identified. These methods permit an evaluation of differences in variables without the risk of problems with mass significance and need for normal distribution of data or strict independence among variables as in conventional statistics. We used PLS-DA to find a model able to separate the three clinical classes: (1) healthy, (2) atopic eczema and respiratory allergy, and (3) isolated respiratory allergy, respectively. The separation is based on all variables analysed, i.e. breast milk levels of fatty acids, lactose and protein, serum phospholipids fatty acids and dietary variables. Multivariate statistical analyses were performed using Simca-P+12.0 (Umetrics, Umeå, Sweden).

The variables identified by PLS-DA as most important for class separation were investigated by univariate statistical analyses comparing the three groups of women. Univariate analyses were performed with SPSS 15.0 (SPSS Inc., Chicago, IL, USA). Mann–Whitney *U*-test was used to determine differences between the subject groups (*P*≤0.05 was considered significant) regarding fatty acids, since these were not normally distributed. Student's *t*-test was used to analyse group differences regarding protein and lactose content in milk and dietary intake. Correlations were analysed by Spearman's rank correlation.

## Results

Milk and serum were obtained from 22 non-allergic and 23 allergic women. Among the allergic women, 15 had both respiratory symptoms (asthma and/or AR) and eczema, while seven had respiratory symptoms only and one had only eczema. The women were divided into three groups: (1) eczema and respiratory allergy (*n*=16). This group included those with both eczema and respiratory symptoms as well as the single individual with eczema only. They all tested positive for specific IgE (>160 IU/mL) against at least one allergen, and five of 16 also had raised levels of total IgE (>100 kU/L). (2) Isolated respiratory allergy (*n*=7), defined as asthma and/or allergic rhinoconjuctivitis, but no eczema. All had specific IgE against at least one allergen and three of seven also had elevated levels of total IgE. (3) Non-allergic (*n*=22) defined as no symptoms and negative tests for specific IgE (<160 IU/mL) against all panel allergens as well as a total serum IgE level below 100 kU/L. The women in the eczema and respiratory allergy group reported allergic reactions against the following: grass (*n*=2), grass and furred pets (*n*=2), grass and foods (*n*=2), furred pets and foods (*n*=3), foods and birch pollen (*n*=1), furred pets, foods and birch pollen (*n*=1), grass, foods and birch pollen (*n*=1), grass, foods, birch pollen and mugwort (*n*=1), grass, furred pets, foods, birch pollen and mugwort (*n*=1) and furred pets, foods, birch pollen and mites (*n*=1). In the isolated respiratory group, women reported allergic reaction against the following: grass and mugwort (*n*=1), foods and birch pollen (*n*=1), furred pets (*n*=1) and birch pollen (*n*=4). Thus, women with eczema and respiratory allergy reported responses to 2.4 allergen groups compared with 1.3 in the ‘respiratory only’ group.

### Multivariate statistical analysis by PLS-DA

**Fig. 2:**
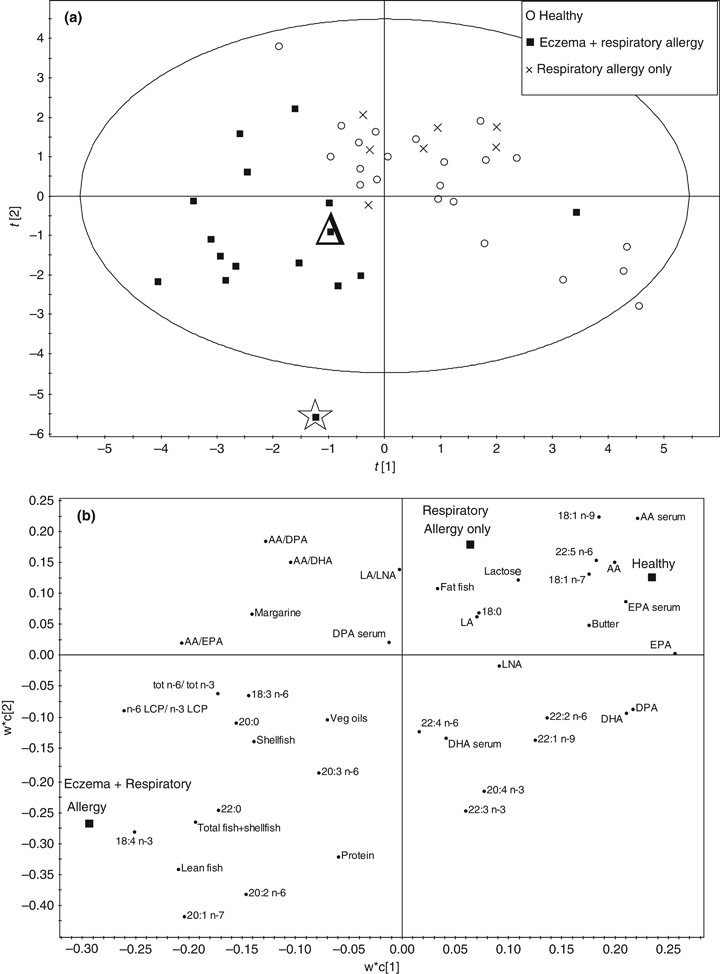

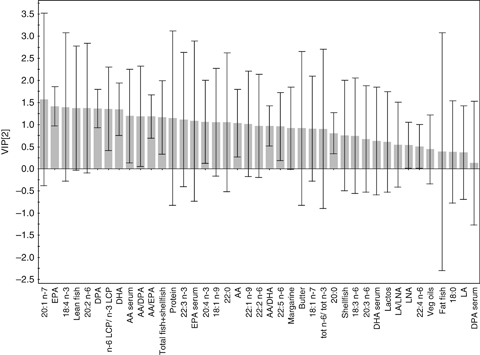
(a) PLS with discriminant analysis (PLS-DA) score plot showing an overview of the separation between the three clinical groups: healthy, eczema plus respiratory allergy or isolated respiratory allergy. Each symbol represents one individual and the position of the symbol is achieved through a combination of this individual's values on all variables. (b) PLS-DA loading plot displaying to what extent the different variables contribute to the class separation. (c) Variables importance in the projection plot showing the degree by which each of the variables contributes to separate the three groups [(Δ=subject taking fish oil supplement, 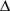
=subject taking flaxseed oil supplement (rich in LNA), LCP=long-chain polyunsaturated fatty acids].

Fatty acids, lactose and proteins were analysed in breast milk and fatty acids in serum. Intake of fat containing food items were registered as estimated intake per week (g/wk). Altogether 41 variables were used to obtain separation between the three clinical groups: healthy, eczema plus respiratory allergy or isolated respiratory allergy. In the PLS-DA score plot (Fig. 2a), each symbol represents one individual and the position of the symbol is achieved through a combination of this individual's values on all variables. Women with both eczema and respiratory allergy were clearly separated from both women with only respiratory allergy and healthy women, while the latter two groups showed almost complete overlap. The PLS-DA loading plot (Fig. 2b) shows to what extent the different variables contribute to the class separation. The loading plot and the score plot correspond; hence variables located in the lower left corner of the loading plot have higher values in subjects located in the lower left corner of the score plot, i.e. those belonging to the eczema and respiratory allergy group, whereas variables in the upper right corner tend to attain higher values in healthy women and women with respiratory symptoms only. High breast milk levels of n-3 fatty acids of the types found in fish, i.e. EPA, DPA and DHA, as well as the n-6 fatty acid arachidonic acid (AA) were all clustered in the ‘healthy’ corner together with high serum levels of EPA (Fig. 2b). Conversely, 18:4 n-3, 20:1 n-7 and 20:2 n-6 as well as high ratio of n-6 to n-3 PUFAs clustered in the lower left corner, close to the ‘eczema and respiratory allergy’ class position. Interestingly, high dietary intake of fatty fish appeared close to the ‘respiratory allergy only’ group, but high consumption of lean fish and total intake of fish and shellfish were connected to the ‘eczema and respiratory allergy’ class. Butter consumption appeared close to the healthy state.

The Variables of Importance in the Projection-plot (VIP-plot), Fig. 2c, shows the degree by which each of the variables contribute to separate the three groups. The higher bar, the stronger discriminatory power of the variable, i.e. the more it contributes to the group separation, and the smaller the variance bars, the greater reliability of its contribution. The loading plot and the VIP-plot together show that among 41 included variables, the milk levels of EPA, DPA and DHA, i.e. fish fatty acids of the n-3 series, had strong discriminatory power in separating the three groups (eczema and respiratory allergy, respiratory symptoms only and healthy) and that high levels were associated with the healthy state. The fatty acids 18:4 n-3, 20:1 n-7 and 20:2 n-6 also had strong discriminating power in the PLS-DA model, but were instead associated with the eczema and respiratory allergy state. Further, serum EPA and AA were predictors of healthy state, whereas high breast milk ratios of AA/EPA, AA/DPA, AA/DHA or long-chain n-6 PUFAs/n-3 PUFAs (n-6 LCP/n-3 LCP) were significant contributors to the model, but with opposite contribution – i.e. being lower in the healthy group. Among the dietary variables, lean fish and total intake of fish and shellfish had strongest discriminatory power in separating the groups. Those were high in the ‘eczema and respiratory allergy’ class.

### Univariate analysis

**Fig. 3:**
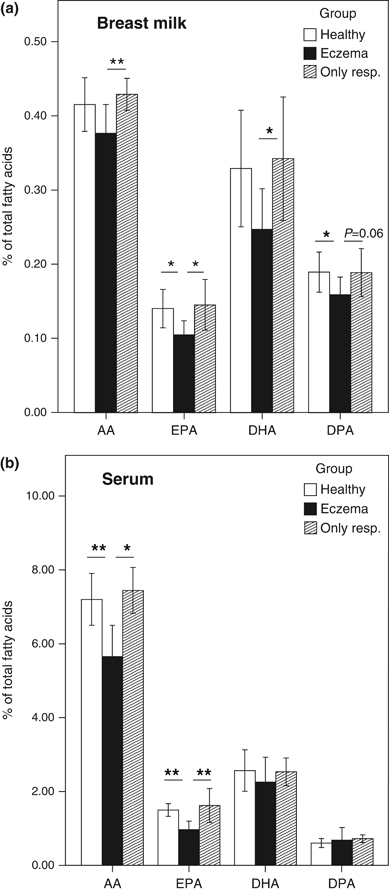
Fatty acids (% of total fatty acid content) in the total lipid fraction of breast milk (a) and serum phospholipids (b) from healthy women, women with eczema and respiratory allergy or respiratory allergy only (^*^P<0.05, ^**^P<0.01).

The results were confirmed by univariate analysis. Women with eczema and respiratory allergy differed clearly from the other two groups having lower breast milk levels of AA, EPA, DHA and DPA than either healthy women or women with isolated respiratory allergy or both (Fig. 3a). In contrast, women with isolated respiratory allergy did not differ from healthy ones (Fig. 3a). Women with eczema and respiratory allergy also had the highest ratio of long-chain n-6 PUFAs/n-3 PUFAs (*P*=0.03 and 0.02 comparing with the ‘healthy’ and ‘isolated respiratory allergy’ groups, respectively) and also higher ratio of AA/EPA (*P*=0.06 and 0.07 compared with the ‘healthy’ and ‘isolated respiratory allergy’ groups, respectively) and total n-6 fatty acids/n-3 fatty acids (*P*=0.09 when comparing healthy and eczema and respiratory allergy groups) in their breast milk, Table 1.

The fatty acids involved in the n-6 and n-3 PUFA metabolic pathways (Fig. 1) correlated positively to one another (data not shown). Protein and lactose concentrations did not differ between the groups of subjects (data not shown).

### Fatty acids in serum phospholipids

Fatty acid composition in serum phospholipids were assayed in blood samples. Women with eczema and respiratory allergy had low serum levels of AA (*P*=0.01 and 0.02 compared ‘healthy’ and ‘isolated respiratory allergy’ groups, respectively) and EPA (*P*=0.001 and 0.007, respectively), Fig. 3b. The levels of AA, EPA, DHA and DPA were similar between the groups of healthy and atopic women with respiratory allergy only.

### Dietary data

The weekly intake of food products expected to affect the levels of long-chain n-3 fatty acids were assessed by food-frequency questionnaire and converted to intake in g/wk. As seen in Table 2, women with eczema and respiratory allergy reported intake of as much fatty fish as healthy women and women with isolated respiratory allergy or even higher intake. Women with eczema and respiratory allergy reported significantly higher lean fish consumption compared with women with isolated respiratory allergy (110 ± 36 and 31 ± 6.3, respectively, *P*=0.05), and also higher total intake of fish and shellfish than healthy women (180 ± 42 and 110 ± 15, respectively, *P*=0.08). Women with respiratory allergy only consumed less butter than did healthy women (5.0 ± 5.0 and 21 ± 3.7, respectively, *P*=0.03).

**Table 2:** Dietary intake of selected fat containing food items

	Intake (g/wk)			
		
	Healthy (*n*=20)	Eczema+respiratory allergy (*n*=14)	Only respiratory allergy (*n*=7)	*P*-value
Fatty fish	39 ± 8.6	38 ± 12	90 ± 50	NS
Lean fish	55 ± 11	110 ± 36^*^	31 ± 6.3^*^	0.05
Shellfish	16.6 ± 2.6	32.1 ± 16.3	16.1 ± 4.1	NS
Total fish+shellfish	110 ± 15^*^	180 ± 42^*^	130 ± 56	NS (*P*=0.08)
Butter	21 ± 3.7^*^	11 ± 4.2	5.0 ± 5.0^*^	0.03
Margarine	11 ± 3.6	18 ± 4.4	17 ± 6.6	NS
Vegetable oils	15 ± 2.7	17 ± 3.8	13 ± 5.9	NS

Data was determined from food frequency questionnaires and converted to g/wk.

Mean ± SEM, Student's *t*-test.

^*^Significant difference between the indicated groups, with *P* value as indicated, NS, not significant, ^*^P=0.08

## Discussion

In this study, we compared fatty acid composition in breast milk and serum samples from atopic and non-atopic women using a multivariate approach that enables comparisons between groups of patients based on a large set of variables. The atopic women had either respiratory allergy only, or a combination of respiratory allergy and eczema, while a single individual had eczema as the only clinical manifestation of the atopic state. The groups with both skin and respiratory symptoms were also allergic to a larger variety of allergens, indicating more extensive disease. Despite relatively small group sizes, differences in fatty acid content in breast milk and serum were readily observed. Women with eczema and respiratory allergy were found to have a breast milk fatty acid profile which deviated significantly from that of healthy women, but also from women with respiratory allergy only. Most notable was their low levels of several long-chain PUFAs of the n-3 series. In contrast, breast milk composition differed little between women with respiratory allergy alone and healthy women.

Fish and shellfish are the major sources of the long-chain n-3 PUFAs EPA, DHA and DPA, and the levels of these fatty acids in serum and breast milk are assumed to reflect intake. However, women with eczema and respiratory allergy reported similar intake of fatty fish and higher weekly intake of marine foods than healthy women. Despite that, women with eczema and respiratory allergy had lower serum levels of EPA, lower concentrations of EPA, DHA and DPA and higher ratios of long-chain n-6 PUFAs/n-3 PUFAs and AA/EPA ratios in the milk samples. Hence, low levels of the long-chain n-3 fatty acids in breast milk of women with eczema and respiratory allergy occurred despite higher fish consumption.

Theoretically, the low levels of n-3 PUFAs in women with eczema and respiratory allergy could depend on dysfunction of enzymes converting linoleic and α-linolenic acid into long-chain n-6 and n-3 PUFAs [18, 37] (Fig. 1). Genetic variations in FADS1 and FADS2, the Δ5- and Δ6-desaturase which carry out this transformation, have been shown to influence maternal plasma and breast milk fatty acids during pregnancy and lactation [38]. It has, indeed, been proposed that FADS gene clusters have an impact on the risk of developing atopic disease [39].

Another explanation which is equally likely is that the comparatively low levels of n-3 fatty acids in allergic women are not due to low intake and/or synthesis, but to enhanced consumption. PUFAs are raw material for synthesis of prostaglandins and leukotrienes which may be consumed during the allergic process. Accordingly, we noted that a dramatic fall in serum levels of EPA, DHA and AA concomitant with challenge in the mouse ovalbumin-asthma model [27]. When comparing women with only respiratory symptoms with healthy women, the only difference found was a slightly higher ratio of total n-6 fatty acids/n-3 fatty acids in milk from atopic women. However, consumption of long-chain n-3 PUFAs cannot be excluded in this group either, because women with isolated respiratory allergy had the highest intake of fatty fish of all groups. The fact that low levels of EPA, DHA and DPA were only seen in the groups of women with both skin and respiratory atopy could be explained by a more extensive disease process, involving more than one organ and a greater number of allergens, compared with the women with isolated respiratory allergy.

Despite what is said above, we cannot exclude that long-chain n-3 PUFAs are protective against skin manifestations, but not respiratory allergy. The etiology and pathogenesis of atopic eczema is complex involving mainly T helper cells producing typical Th2 cytokines, such as IL-4 in early lesions, but dominated by the Th1 cytokine IFN-γ in the chronic phase [40]. Long-chain n-3 PUFAs are known to down-regulate inflammation. In fact, we have observed in mouse models of Th1 and Th2 hypersensitivity that dietary fish oil reduces Th1 sensitization in a skin DTH model, but rather increased Th2 [27]. The immunomodulatory mechanisms of n-3 fatty acids can only be speculated upon. But n-3 PUFAs affect production of prostaglandins and related mediators, fluidity in membranes and gene transcription [41]. Intake of n-3 PUFAs interfere with thrombocyte activation and coagulation [42], which are intimately linked to inflammation and immunity, especially Th1-driven immune reactions [43]. If fish oil is protective and active against the Th1-driven phase of eczema, it may explain why women with eczema and respiratory allergy have lower serum and breast milk levels of n-3 fatty acids than both women with only respiratory allergy and healthy women.

Interestingly, the original theory proposed by Dunder [4], that high margarine consumption and low butter consumption may predispose to allergy was supported by the findings of our study. Thus, low butter consumption characterized allergic women compared with healthy ones. Whether this is the result of a wish for more ‘healthy’ life-style, or whether this diet actually aggravates allergy cannot be deduced from our study.

Most studies investigating associations between allergy and n-3 fatty acids in breast milk have included atopic subjects with different types of allergic diseases in the same group. The contradictory results reached in these studies may, thus, not only depend on factors as season, duration of lactation and maternal diet as proposed [44], but might also depend on the allergic manifestation examined. Furthermore, different groups may differ in intake as suggested here. Hence, if respiratory allergy is prevalent among the examined subjects, fewer differences in fatty acid pattern may be expected to appear, than if a high proportion of subjects with eczema are included. The finding of low n-3 fatty acid levels in subjects with eczema and respiratory allergy corresponds to recent reports on low serum levels of EPA and DHA in children with atopic dermatitis [4], the association between low rate of eczema and early fish introduction in children's diet [7–9], and that breast milk given to infants who later developed atopic eczema has low levels of n-3 fatty acids [19].

Proportionally high levels of the fatty acids 18:4 n-3, 20:1 n-7 and 20:2 n-6 were found in the breast milk of women with eczema and respiratory allergy. Whether this is a secondary phenomenon or important in the pathogenesis of this disease cannot be deduced from our study, as very little is known about their potential physiological effects. 18:4 n-3, stearidonic acid, is a precursor of EPA and is suggested to have similar biological effects and functions as EPA [45]. It is found in seafood [46] and the high levels in women with eczema and respiratory allergy might relate to their high fish intake. Less is known about the occurrence of 20:1 n-7 and 20:2 n-6 in different kinds of food.

Our results show that low breast milk and serum levels of long-chain n-3 fatty acids are associated with extensive allergic disease including eczema, and that this is not due to low fish intake. We suggest that long-chain n-3 fatty acids are consumed during allergic inflammation as the most likely explanation. However, this does not exclude that n-3 PUFAs may be protective against immune effector functions important in eczema but not in respiratory allergy, e.g. the strong Th1 component of the chronic eczematous phase. Our results clearly show the importance of defining the type of allergic disease involved before giving dietary advice or testing n-3 fatty acid supplementation for disease prevention or treatment.
